# Brain metastases in ALK-positive NSCLC – time to adjust current treatment algorithms

**DOI:** 10.18632/oncotarget.26073

**Published:** 2018-10-12

**Authors:** Frank Griesinger, Julia Roeper, Christoph Pöttgen, Kay C. Willborn, Wilfried E.E. Eberhardt

**Affiliations:** ^1^ Department of Hematology and Oncology, University Department Internal Medicine-Oncology, Pius-Hospital, Medical Campus University of Oldenburg, Oldenburg, Germany; ^2^ Department of Radiotherapy, University Hospital Essen, Essen, Germany; ^3^ Department of Radiotherapy and Radiooncology, University Department of Medical Physics, Pius-Hospital Oldenburg, University of Oldenburg, Oldenburg, Germany; ^4^ Department of Medical Oncology, West German Cancer Center, Ruhrlandklinik, University of Duisburg-Essen, Essen, Germany

**Keywords:** non-small cell lung cancer, ALK-positive, brain metastases, ALK-inhibitors

## Abstract

The progress in molecular biology has revolutionized systemic treatment of advanced non-small-cell lung cancer (NSCLC) from conventional chemotherapy to a treatment stratified by histology and genetic aberrations. Tumors harboring a translocation of the anaplastic-lymphoma-kinase (ALK) gene constitute a distinct genetic and clinico-pathologic NSCLC subtype with patients with ALK-positive disease being at a higher risk for developing brain metastases. Due to the introduction of effective targeted therapy with ALK-inhibitors, today, patients with advanced ALK-positive NSCLC achieve high overall response rates and remain progression-free for long time intervals. Moreover, ALK-inhibitors seem to exhibit efficacy in the treatment of brain metastases. In the light of this, it needs to be discussed how treatment algorithms for managing patients with brain metastases should be modified. By integrating systemic ALK-inhibitor therapy, radiotherapy, in particular whole brain radiotherapy might be postponed deferring potential long-term impairment by neurocognitive deficits to a later time point in the course of the disease. An early treatment of asymptomatic brain metastases might offer patients a longer time without impairment of cerebral symptoms or radiotherapeutic interventions. Based on an updated extensive review of the literature this article provides an overview on the epidemiology and the treatment of patients’ brain metastases. It describes the specifics of ALK-positive disease and proposes an algorithm for the treatment of patients with advanced ALK-positive NSCLC and brain metastases.

## INTRODUCTION

Lung cancer remains one of the major challenges in oncology. It is the most frequent cause of cancer death worldwide [1, 2, 3]. In Germany, it is the second most frequent newly diagnosed malignant disease in men after prostate cancer, and the third most frequent in women after breast and colon cancer. In 2012, according to the most recent numbers of the Robert-Koch-Institute, 34,490 men and 18,030 women were diagnosed in Germany. Lung cancer was the leading cause of cancer death in men with 29,713 deaths (25%) and the second most frequent cause of cancer death in women with 14,752 deaths (15%). Five-year overall survival rates were 16% for men and 21% for women [4]. According to the American Cancer Society non-small-cell lung cancer (NCSCLC) is the most common type and accounts for about 85% of all lung cancers. Squamous-cell carcinoma (25-30%), adenocarcinoma (40%) and large-cell carcinoma (10-15%) all are subtypes of NCSLC [82].

Treatment of patients with non-small-cell lung cancer (NSCLC) is guided by disease stage. Early stages and some of the locally advanced stages are treated with a curative intent. Surgery, radiation, primary (neoadjuvant) and adjuvant chemotherapy are the respective treatment options, mostly as one component of combined multimodality therapy [5, 6]. Treating patients with stage IV disease represents a palliative setting in which improvement of symptoms, retaining or even improving quality of life and prolonging overall survival are relevant treatment objectives [5, 6]. Oligometastatic disease (OMD) may represent a potentially curative situation as long as there is only a limited involvement of mediastinal lymph nodes [83].

Over the last 15 years medical research and, in particular, the progress in molecular biology has fundamentally changed our understanding of lung cancer. Meanwhile we know that the genotype of the tumor is an important prognostic and in some cases predictive factor besides the classical clinico-pathologic factors such as disease stage, histology, gender, performance status or comorbidity. Moreover, the progress in molecular biology revolutionized systemic treatment of advanced NSCLC from chemotherapy to a treatment stratified by histology and genetic aberrations consisting of monoclonal antibodies, a panel of targeted kinase-inhibitors and chemotherapy [5, 6]. All NSCLC patients with a non-squamous histology and never or light smokers (< 10 pack years and > 15 years from smoking cessation) with squamous-cell carcinoma should be screened for EGFR mutations and for ALK- and ROS1 translocations before starting a systemic first-line therapy [6, 54].

### ALK-positive NSCLC

Tumors harboring a translocation of the anaplastic-lymphoma-kinase (ALK) gene constitute a distinct genetic and clinico-pathologic NSCLC subtype. An inversion on the short arm of chromosome 2 results in a fusion of the ALK-gene with the „echinoderma microtubule-associated protein-like 4“ (EML4)-gene. Transcription of this newly formed oncogene results in the production of the fusion protein EML4-ALK. By activation of subsequent signal transduction cascades, the fusion protein leads to cell proliferation, inhibition of apoptosis and ultimately to the stimulation of tumor growth. This particular genetic NSCLC subtype was initially described by Soda and colleagues [7]. Since then, a number of EML4-ALK-variants [8, 9, 7, 10, 11, 12] and ALK fusion proteins with alternative fusion partners other than EML4 have been discovered [11, 13]. An ALK-translocation is detected in 3-7% of all NSCLC patients [7, 9, 14, 15, 10, 12, 16]. Their tumors rarely exhibit simultaneous mutations of EGFR or KRAS [17], in contrast to EGFR mutations, ALK translocations do not seem to be dependent on ethnicity.

ALK-positive NSCLC is not only a genetic subtype but also a clinical entity, ie patients having this tumors do carry specific clinical characteristics. ALK-positive tumors are mainly, but not exclusively associated with adenocarcinoma histology. Patients with ALK-positive NSCLC more commonly are never- or light smokers, and they have a median age of between 50 and 55 years, which is about 10 to 15 years lower than for the general NSCLC population and also the EGFR mutated patients [9, 13, 18, 19, 20, 21, 22, 23, 84].

While mutations of the EGFR gene represent a prognostic and a predictive marker, an ALK translocation is mainly a predictive marker, as the prognosis of patients not treated with ALK-specific tyrosine kinase inhibitors (TKI) is as unfavorable as the prognosis of NSCLC patients with no detectable EGFR or ALK gene mutation (wild-type (WT) NSCLC). Inhibition of the ALK tyrosine kinase and its subsequent signal transduction pathway with specific TKI's results in tumor growth arrest, improved clinical response and a survival benefit in ALK-positive NSCLC as compared to WT NSCLC [24, 25, 26, 27, 28].

### Brain metastases

Brain metastases are a relevant problem in NSCLC. Up to 64% of all patients with lung cancer develop brain metastases during the course of the disease [5, 29]. Numbers for the incidence of brain metastases in NSCLC are available from big patient populations. The incidence was 23.2% in a retrospective study with 1.127 consecutive patients [30]. 17% of 5.133 NSCLC patients documented in a US tumor registry were diagnosed with brain metastases during the course of their disease [31]. A retrospective study with 482 consecutive stage IIIB/IV NSCLC patients reported an incidence of <36% over the course of the disease [32]. A retrospective evaluation of 975 consecutive stage I-II NSCLC patients with curatively intended surgery found the 5-year risk for metastasizing to the brain to be as high as 10% [33]. In the prospective German registry CRISP including all-comers NSCLC receiving systemic therapy, the incidence of brain metastases is 18% (unpublished data, Griesinger et al).

Age < 60 years and adenocarcinoma histology are risk factors for developing brain metastases [34, 35, 33, 36, 37, 38]. According to a retrospective study in 629 patients, the incidence of brain metastases for NSCLC patients with adenocarcinoma amounted to 26.9% [39]. Another retrospective study in 234 NSCLC patients with adenocarcinoma reported an incidence of 32.5% [40]. A cohort study from Israel found 23 of 252 patients with metastatic NSCLC to be ALK-positive. The cumulative incidence of brain metastasis was 23.8% initially and 23.8%, 45.5% and 58.4% after 1, 2 and 3 years, respectively [41]. This indicates, that the longer the patients live the more likely they will develop brain metastases. According to Johung et al. 30% of an ALK-pos. NSCLC collective had brain metastases at the time of diagnosis. All other patients developed brain metastases with a median time of 27 months (range: 2-174) since first diagnosis [64]. Table 1 summarizes landmark data on the development of brain metastases in stage IIIB/IV NSCLC patients.

**Table 1:** Landmark data on the development of brain metastases for patients with stage IIIB/IV

Author	Population	Landmark data for brain metastases (BM)
Alsan Cetin I et al. 2013 [78]	NSCLC Stage IIIA/IIIB N=200	Incidence of BM after 2 years 23%
Gaspar LE et al. 2005 [36]	NSCLC Stage IIIA/B and chemotherapy N=421 N=71 with BM	Onset of BM 22.5% during therapy, 24% 0-16 weeks after therapy, 14% 16 weeks - 6 months after therapy. 22.5% 6-12 months after therapy, 17% > 12 months after therapy
Boggs DH et al. 2014 [79]	NSCLC Stage IIIA, IIIB, IV without BM and no PD N=45	Incidence of BM after 1 year 13% without and 18% with temozomolide
Liu J et al. ASCO 2013 [94]	Wt NSCLC Stage III/IV without BM N=72	Median time to onset of BM 19.0 months Incidence after 1 year 21.1%, after 2 years 50.2%
Hsiao et al. 2013 [32]	NSCLC Stage IIIB/IV N=482	Incidence of BM 42% after 3 months, 54% after 1 year, 64% after 2 years
Arieta O et al. 2009 [80]	NSCLC Stage IIIB/IV N=293	Incidence of BM 27% after 1 year, 32% after 2 years Incidence of BM in patients with adenocarcinoma 16.4% after 1 year, 20,2% after 2 years
Hendriks LE et al. 2014 [81]	Wt NSCLC Stage IV N=62	Mean time to onset of BM 10.7 months
Rangachari D et al. 2015 [41]	ALK-pos. NSCLC 91,3% Stage IV N= 23	Cumulative incidence of BM 23.8% bei Erstdiagnose, 23.8% after 1 year, 45.5% after 2 years, 58.4% after 3 years
Chua D et al. 2010 [69]	NSCLC and ≥ 1 BM N=47 with WBRT + temozomolide N= 48 with WBI	Median time to CNS progression 3.1 months with WBI + temozomolide and 3.8 months with WBI
Han G et al. ASTRO 2015 [40]	NSCLC without BM at primary diagnosis N=195	Cumulative incidence of BM 4.2% after 1 year, 18.7% after 2 years for wt NSCLC
Duma N et al. ASCO 2015 [53]	NSCLC N=172	Median time from primary diagnosis to onset of BM 259 days

*Data on EGFR-positive NSCLC or EGFR-inhibitor treated patients were not incorporated.

Patients with ALK-positive NSCLC seem to be at higher risk for developing brain metastases [42, 43]. However, epidemiologic data are still rare. The analysis of three databases comprising a total of 1,352,449 lung cancer patients from US routine clinical practice found 947 patients with ALK-positive disease. Of these, 28% had brain metastases diagnosed with a median of 88 days after primary diagnosis [44]. There are also numbers from clinical trials which can, however, only serve as rough estimates due to the selected patient populations treated within clinical trials. In the phase-III study PROFILE 1014 that compared crizotinib as first-line therapy against chemotherapy, 27% of patients had brain metastases at baseline [45]. 40% of patients in the phase-III study ALEX comparing alectinib vs. crizotinib as first-line therapy showed brain metastases at baseline [86]. In the phase-III Study ASCEND 4 comparing in first line ceritinib vs. chemotherapy (pemetrexed and cis- or carboplatin) in ALK+ NSCLC, 32% of patients had brain metastases at diagnosis. Although these different studies had different inclusion criteria (treated brain metastases only in the crizotinib trial, asymptomatic metastases with or without irradiation in the ceritinib and alectinib trials), the rates of CNS metastases at diagnosis were comparable. In the second line setting, the PROFILE 1007 study assessing crizotinib versus chemotherapy after prior platinum (ALK-inhibitor naïve patients), the proportion of patients with brain metastases was 35% [24]. In the phase-I study ASCEND-1, in which ALK-inhibitor naïve and ALK-inhibitor pretreated patients received ceritinib, 31% of ALK-inhibitor naïve and 60% of ALK-inhibitor pretreated patients had brain metastases [47]. In the phase-II study ASCEND-3 ALK-inhibitor naïve patients with up to 3 prior lines of chemotherapy received ceritinib, 39.5% of whom had brain metastases at inclusion in the study [48]. In the phase-II study ASCEND-2, 71.4% of patients progressing during or after crizotinib had brain metastases [49]. In the phase-III study ASCEND-5 comparing chemotherapy vs. ceritinib in crizotinib-pretreated patients, 60% and 57% had brain metastases at baseline [50]. In the randomized phase-II trial ALTA comparing two doses of brigatinib in crizotinib-resistant patients, 69% had brain metastases at baseline [51]. The proportion of patients with brain metastases in both phase-II trials testing alectinib after prior crizotinib was 60% at baseline [28, 26].

A number of factors contribute to the detection of brain metastases among them disease stage, subtype, tumor biology and prior therapies but also the diagnostic methods and intervals [5, 52]. A retrospective study with 1,602 NSCLC patients treated between 2000 and 2013 reported a median time of 259 days (8.5 months) from primary diagnosis to the onset of brain metastases. It was not specified whether the metastases had been diagnosed by imaging diagnostics or due to the emergence of symptoms [53]. Another study found a median time of 14.3 months for the onset of brain metastases in NSCLC patients with adenocarcinoma when they were diagnosed clinically [39].

Currently, screening for brain metastases is not recommended as a routine follow-up measure for asymptomatic patients [5, 54]. The extensive literature search performed as the basis for this review article did not retrieve data documenting at which point in time NSCLC patients and, in particular, patients with ALK-positive NSCLC with initially asymptomatic brain metastases become symptomatic. It is, however, only a question of time until brain metastases grow to a critical size. The size of brain metastases from NSCLC of 19 minimally symptomatic patients was documented by serial magnetic resonance imaging (MRI) during and after first-line chemotherapy. Tumor volume increased on average by 1.7% per day and doubled after a median of 58.5 days [55].

Patients with brain metastases are compromised by headaches, signs of increased intracranial pressure, focal neurological signs (hemiparesis, aphasia, ataxia, vision disorders or brain stem symptoms), epileptic seizures or neurocognitive deficits [5, 56, 57, 58]. Impairments of neurocognitive function depend on the size and location of the brain lesions and the surrounding perifocal edema. A study of 401 patients with whole brain radiotherapy (WBRT) including 251 NSCLC patients found 21-63% to have some kind of impaired neurocognitive function at baseline. Patients progressing 2 months after WBRT had a deterioration of neurocognitive function compared to baseline. The study found an improvement in two domains of neurocognitive function if imaging showed a partial response 2 months after WBRT [58].

Studies documenting the follow-up of curatively resected NSCLC patients show that those with asymptomatic recurrences have a survival benefit compared to patients with symptomatic recurrences [59, 60]. It is, however, unclear whether being symptomatic or not does influence the prognosis of patients with brain metastases. A retrospective study documented symptoms in 46.7% of patients with brain metastases from lung cancer. Overall brain metastases were detected in 31.3% (n=61) of patients by MRI which was an obligatory staging measure. No differences in size and localization of metastases, presence of perifocal edema, hemorrhage or necrosis were noticed between neurologically symptomatic and asymptomatic patients. Also, tumor load in asymptomatic patients was comparable to that of patients with symptomatic brain metastases [61]. Another series of 183 newly diagnosed NSCLC patients who had MRI screening did not find differences in overall survival (OS) or 1-year survival between patients with symptomatic (n=7) and asymptomatic (n=38) brain metastases [52]. A further retrospective, monocentric study compared neurologically asymptomatic (n=12) and symptomatic NSCLC (n=69) patients with brain metastases and found longer OS for the asymptomatic group (median OS 7.5 vs. 4 months). A higher rate of patients from the asymptomatic group did not develop any neurologic signs in the further course of therapy. They identified an active treatment i.e. surgery, chemotherapy and/or radiotherapy as the strongest prognostic factor and concluded that patients benefit from early treatment of brain metastases [62].

Overall prognosis of NSCLC patients with central nervous system (CNS) metastases is poor. A study with 1,833 patients reported a median OS of 7 months [63]. Patients with ALK-positive NSCLC and brain metastases may have a more favorable prognosis presumably due to treatment with ALK-inhibitors. This is suggested by a retrospective study in 90 ALK-positive NSCLC patients treated between 2007 and 2014 in 6 US centers. About 30% of patients had brain metastases already at primary diagnosis, 80% were younger than 60 years, 47% had more than 3 metastases, 83% were stage IIIB or IV, 70% had additional extracranial metastases. Median OS after the onset of brain metastases was 49.5 months and median PFS 11.9 months in this group of patients characterized by rather unfavorable prognostic factors. 84 patients had been treated with crizotinib, 41 with ceritinib. Repeated radiotherapy interventions were common. 45% of patients had progressive brain metastases at the time of their death [64].

### Treatment of brain metastases

Treatment of brain metastases depends on the number of lesions, location and on the patient's performance status and mostly involves radiotherapy. In case of a solitary lesion, local treatment by surgical resection or stereotactic radiotherapy may suffice and whole brain radiotherapy (WBRT) may be postponed until recurrence or progression. A similar strategy can be followed for patients in a good performance status with 2- 4 brain metastases. In that situation postponement of WBRT may only be an option for individual patients in order to prevent radiotherapy associated side effects. However, one should keep in mind that while adjuvant WBRT has an effect on local tumor control, it has no proven impact on overall survival [54, 65, 66]. WBRT is the treatment of choice for multiple brain lesions [5]. Roughly 40% of patients respond [67, 58]. Median times for overall survival and time to CNS progression are about 4 months and 3 months, respectively [68]. In case of a very poor performance status and an unfavorable prognosis treatment may be limited to symptomatic steroid therapy. Data from a current phase-III trial in 538 NSCLC patients and inoperable brain metastases showed no benefit for WBRT over supportive therapy with dexamethasone only. There was no significant difference in quality of life between the two treatment arms [69].

Systemic treatment with drugs crossing the blood-brain barrier is recommended for patients with asymptomatic or only minor symptomatic brain metastases. For those patients, radiotherapy is not generally indicated upfront but represents a therapeutic option in case of further progress [54].

Radiotherapy is associated with short- and long-term side effects. It may lead to acute exacerbation of peritumoral edema resulting in a transient, yet further deterioration of neurologic symptoms. Radiation-induced necrosis, encephalopathy, vascular damages or demyelination may occur 6 months after WBRT, in many cases irreversible and progressive. They may constitute solely radiographic findings or can result in neurocognitive impairment [56]. There is evidence that WBRT is associated with an increased risk of long-term deterioration of neurocognitive functions [70, 71]. This has been demonstrated by a study in which patients with a maximum of 3 CNS metastases with a diameter not exceeding 3 cm either received stereotactic therapy alone or in combination with WBRT. Survivors that had been treated with WBRT showed a worsening of neurocognitive functions [71]. Data from an RTOG-study suggest that WBRT sparing the hippocampal region may result in fewer neurocognitive deficits [72].

### ALK-inhibitor therapy

The situation of patients with ALK-positive NSCLC has changed dramatically since the introduction of effective targeted therapy with ALK-inhibitors. Today, patients with advanced ALK-positive NSCLC achieve high overall response rates and remain progression-free for long time intervals. After prior platinum therapy, crizotinib resulted in ORR of 65%, median PFS of 7.7 months and median OS of 21.7 months [26]. First-line therapy with crizotinib achieved ORR of 74% and a median PFS of 10.9 months [45]. A phase-I study of ceritinib in 163 patients with advanced NSCLC and prior crizotinib showed an ORR of 56%, median duration of response of 8.3 months and median PFS of 6.9 months. In those 83 patients without prior ALK-inhibitor treatment, ORR was 72%, median duration of response 17 months and median PFS 18.4 months, respectively [25, 47]. These data were confirmed by the first-line study with ceritinib (ASCEND 4) which showed a significantly different ORR, PFS and DCR in ALK+ tumors in comparison with chemotherapy (HR for PFS: 0.55, 95% CI 0.42-0.73) [87]. In the phase-II study ASCEND-3, the ORR with ceritinib was 63.7% and median PFS 18.4 months in 124 ALK-inhibitor-naive patients with up to 3 prior lines of chemotherapy [48]. The phase-III ASCEND-5 in 231 crizotinib-pretreated patients compared chemotherapy vs. ceritinib and found a median PFS of 1.6 vs. 5.4 months (HR 0.49) and ORR of 7% vs. 39% [50].

A randomized phase-II study comparing two doses of brigatinib (90 mg qd and 180 mg qd) in 222 crizotinib-resistant patients reported ORR of 45% and 54% and median PFS of 9.2 months and 12.9 months, respectively [51].

A recent Japanese phase-III study in ALK-inihibitor naïve patients showed an ORR of 78.9% for crizotinib and 91.6% for alectinib. First-line treatment with alectinib resulted in a significant PFS benefit (HR 0.34; p<0.0001). Median PFS with crizotinib was 10.2 months (95% CI 8.2-12.0) while it had not been reached after 24 months with alectinib (95% CI 20.3-NR) [46]. In the Phase III ALEX trial comparing Crizotinib to Alectinib in ALKi naïve patients, median progression free survival with crizotinib was 11.1 months (95% CI 9.1-13.1), whereas median PFS with alectinib was not reached (95% CI 17.7-n.e.). Hazard ratio for disease progression or death was 0.47 (95%CI, 0.34-0.65) p<0.001). 12-month progression free survival rate was 68.4% (95%CI 61.0-75.9) in patients treated with alectinib and in 48.7% (95%CI 40.4-56.9) of patients treated with crizotinib [86].

Moreover, ALK-inhibitors exhibit efficacy in the treatment of brain metastases. The ALK-inhibitor alectinib seems to cross the blood-brain barrier to a relevant extent and, unlike crizotinib and ceritinib, is not a substrate for P-gp (p-glycoprotein). Therefore, alectinib is not actively eliminated from CNS tissue by efflux-mechanisms either. That is supported by preclinical data in mouse models with EML4-ALK-positive NSCLC showing a high brain-to-plasma ratio of alectinib [73] and by a phase-I study that detected relevant concentrations of alectinib in the cerebrospinal fluid of patients with brain metastases [74].

The first-line crizotinib study PROFILE 1014 allowed inclusion of patients with brain metastases if they had been treated and were neurologically stable without corticosteroid medication. Rate of intracranial disease control (stable disease, partial and complete response) in 39 patients with brain metastases was 85% after 12 weeks, 56% after 24 weeks and median time to intracranial disease progression was 15.7 months [27]. After prior platinum treatment, the intracranial disease control rate was 52% in patients with asymptomatic pre-irradiated brain metastases and 56% in patients without prior radiotherapy 12 weeks after crizotinib. The median time to intracranial tumor progression was 13.3 months and 7.0 months, respectively [75].

A retrospective analysis of 94 patients with brain metastases treated with ceritinib in the multicenter phase-1 study ASCEND-1 found a median time to intracranial response of 6.1 weeks. The rate of intracranial disease control was 79% in ALK-inhibitor naïve patients and 61% in those pretreated with ALK-inhibitors. There was no difference in intracranial response in patients with or without prior radiotherapy [47]. In the phase-II study ASCEND-2 including patients progressing during or after crizotinib, intracranial response rate for 20 patients with active brain metastases (newly diagnosed or progressive) was 45% and the rate of intracranial disease control 80% [49]. ASCEND-3 reported rates of 61% for intracranial response and 76.9% for intracranial disease control. Systemic response in patients with brain metastases was poorer than in those without with an ORR of 57.1% and a median PFS of 10.8 months [48]. Chemotherapy and crizotinib pre-treated patients with brain metastases at baseline in ASCEND -5 had a median PFS of 4.4 months (3.5 - 6.2) versus 1.5 months (1.3 – 1.8) in the chemotherapy comparator group [HR 0.54 (95% CI 0.36 – 0.80)] [88]. The intracranial clinical benefit rate in patients with measurable baseline brain metastases in the first-line trial ASCEND-4 for ceritinib at ≥12 weeks as well as ≥ 24 weeks was 86.4% (95% CI, 65.1-97.1) compared with 68.2% (95%CI, 45.1 – 86.1) and 50% (95%CI, 28.2-71.8) for chemotherapy respectively [87].

The randomized phase-II study comparing two doses of brigatinib (90 mg qd and 180 mg qd) reported rates for intracranial response of 36% and 67% and for intracranial disease control of 88% and 83%, respectively, in crizotinib-resistant patients with active brain metastases-[51]. Independent review committee-assessed intracranial ORR in patients with measurable brain metastases at baseline was 42% (11 of 26 patients) for the 90 mg qd-dosing and 67% (12 of 18 patients) for the 180mg qd-dosing. The median intracranial PFS was 15.6 months (95% CI, 7.3 to 15.7) and 12.8 months (95%CI, 11.0 to not reached) in the 90 mg and the 180mg dose, respectively [89].

Treatment of 84 crizotinib-resistant patients with brain metastases with alectinib resulted in a rate of intracranial disease control of 84.5% with 31% of all patients actually achieving a complete response. Median duration of CNS-response was 11.2 months [91]. Another phase-II study of alectinib in crizotinib-pretreated patients showed a disease control rate of 89% and complete response rate of 63% in all and 56% in radiotherapy-naive patients. The median duration of response was 11.1 months [26]. A pooled analysis of these two phase-II trials with alectinib found a lower risk of CNS progression if patients had no brain metastases before starting treatment with alectinib compared to those with a diagnosis of brain metastases at baseline. The radiotherapy-naïve patients of the latter group had a lower probability of CNS progression [76]. In the ALUR trial, 107 patients were randomized to receive alectinib (n = 72) or 2^nd^ line chemotherapy (pemetrexed or docetaxel, n=35) after failure of one platinum doublet chemotherapy and crizotinib. The primary study endpoint of PFS was reached with a HR of 0.15 by investigator assessment. The CNS response rate of patients with measurable brain metastases treated with alectinib was 54.2% and with chemotherapy it was 0% [92]. Within the multinational phase-III study ALEX the rates of CNS response in first-line patients with ALK-positive NSCLC and measurable CNS lesions at baseline for crizotinib were 50% (95% CI 28-72) compared to a CNS response rate of 81% (95% CI 58-95) with alectinib, resulting in a cause-specific hazard ratio of 0.16 (95%CI 0.10-0.28). The median duration of CNS response was 5.5 months (2.1-17.3) with crizotinib and 17.3 months (95%CI: 14.8 to not estimable) with alectinib. 29 patients (45%) of the alectinib group showed a complete CNS response, as compared to 5 patients (9%) in the crizotinib comparator group [86]. Furthermore, the cause specific HR for time to progression in the brain in pts with brain mets was 0.18 for alectinib vs. crizotinib and it was 0.14 for pts without brain metastases. These results indicate a preventive effect of alectinib against the (re-)occurrence of brain mets in patients with and without brain mets [93]. This is supported by the Japanese J-ALEX trial - also comparing alectinib to crizotinib - describing a HR for the time to progression of brain metastases or death for patients with CNS metastases at baseline with 0.16 (0.02-1.28). The hazard ratio for the development of brain metastases or death in patients without brain metastases at baseline was 0.41 (0.17-1.01) for alectinib as compared to crizotinib [85]. This, for the first time - since the introduction of prophylactic cranial irradiation in small-cell lung cancer - demonstrates a prophylactic effect for these patients by the administration of a systemic treatment.

### Implications for the treatment of patients with ALK-positive NSCLC and brain metastases

Treating brain metastases represents an important challenge in the management of patients with ALK-positive NSCLC. ALK-inhibitor therapy results in high overall response rates and in long progression-free intervals. Yet, eventually, patients will progress, often due to secondary mutations or induction of other oncogenic drivers [90] and develop brain metastases. A considerable proportion of patients namely around 30% according recently published data [64] and baseline characteristics from clinical trials in 1^st^ line treatment of ALK-positive NSCLC patients [86, 87, 27] already has a diagnosis of brain metastases before the start of ALK-inhibitor therapy. Registry data suggest that patients with brain metastases and ALK-positive NSCLC have a more favorable prognosis than patients whose tumors do not harbor an ALK-translocation. ALK-positive patients can be treated with several consecutive therapies. Thus, it is possible to effectively manage patients better over a considerably longer period of time. In the light of this, potential long-term side effects of radiotherapy become more important than they were when overall survival of patients was significantly shorter. The therapeutic strategy regarding brain metastases in ALK-positive NSCLC is already changing.

Indications for surgical interventions and stereotactic radiotherapy while postponing WBRT have become wider. WBRT can be planned sparing cerebral regions critical for neurocognitive functioning e.g. hippocampal-sparing whole-brain radiotherapy [72]. Given the high efficacy of ALK-inhibitors in brain metastases, modification of treatment algorithms by integration of systemic ALK-inhibitor therapy needs to be discussed. Postponing radiotherapy might defer potential long-term impairment by neurocognitive deficits to a later time point in the course of the disease. An early treatment of asymptomatic brain metastases could give patients a longer time without symptom impairment or radiotherapeutic interventions. The current recommendations against a general screening for brain metastases as part of the follow-up diagnostics for neurologically asymptomatic NSCLC patients should be discussed. It is fair to consider recommending regular MRI controls after defined intervals of 3-6 months at least for ALK-positive patients as already recommended for patients with advanced ALK-positive NSCLC by the German DGHO-Guideline for diagnosis and treatment of NSCLC. The same applies to the timely detection of further progression of brain metastases under ALK-inhibitor therapy. The goal should be to start radiotherapy in time and avoid potential risks of a delayed start as described for patients with EGFR-mutated lung cancer and respective treatment sequences [77].

**Figure 1:**
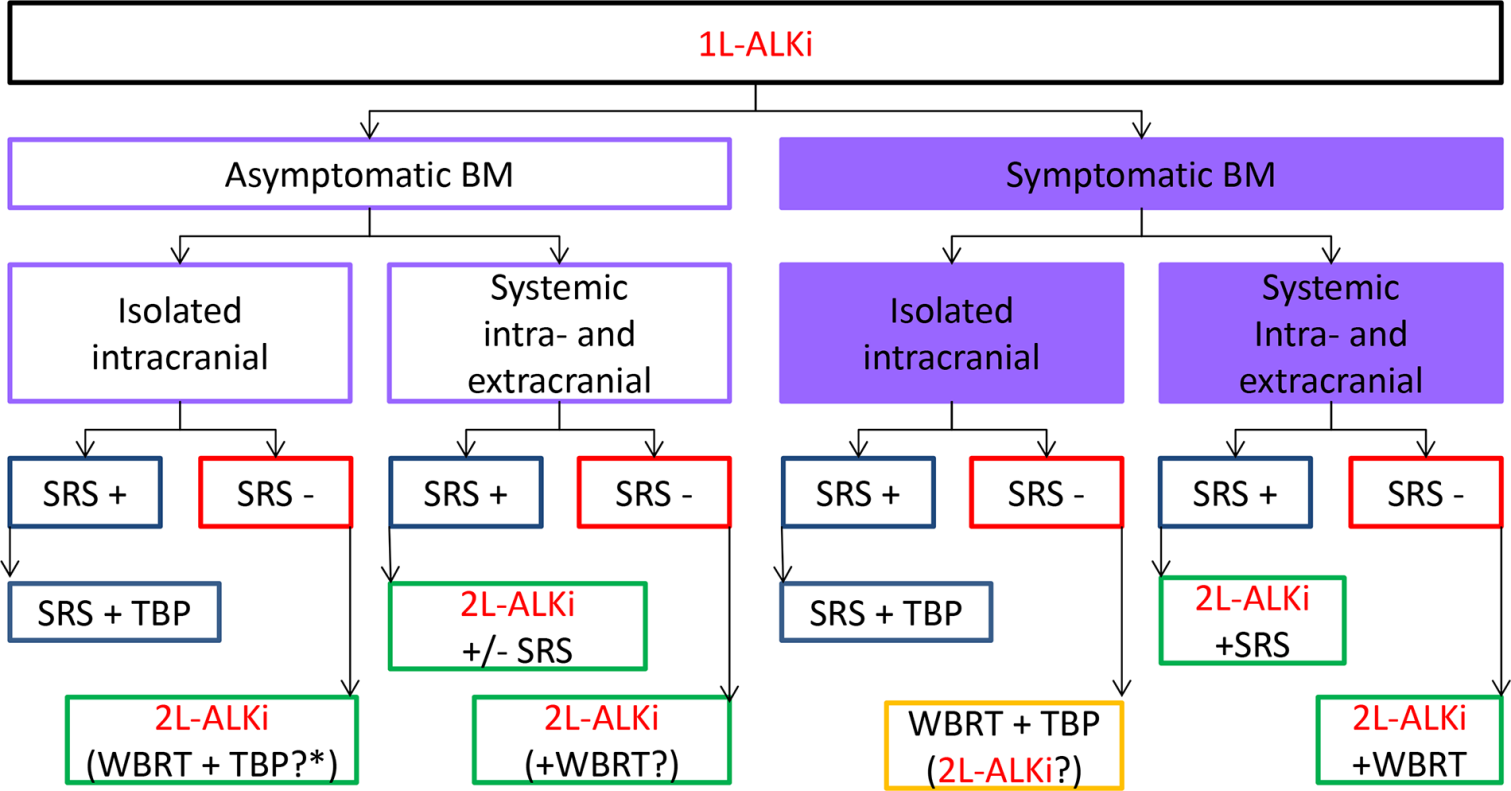

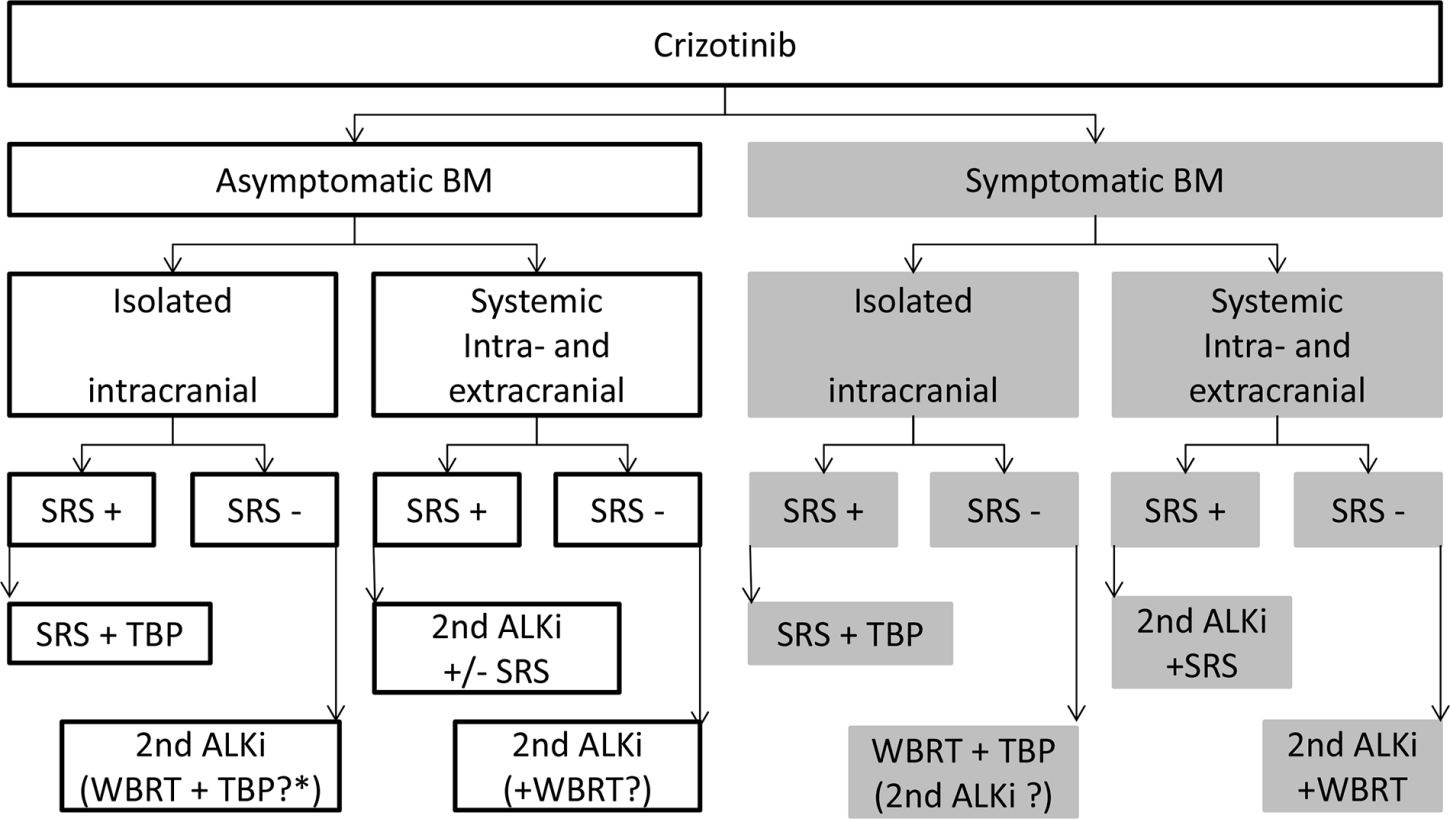
**(A)** Proposed algorithm for the management of patients with ALK-positive NSCLC and brain metastases under treatment with 1L-ALKi. Postpone WBRT as long as possible. If BM not amenable for SRS switch to 2L-ALKi or if sparing WBI (2×20 Gy and/or hippocampal sparing) and TBP if later in the course of the disease. In case mechanism of resistance to 1L-ALKi is known, switch to appropriate 2l-ALKi if possible. BM brain metastases; SRS stereotactic radiotherapy; WBRT whole brain radiotherapy; TBP treatment beyond progression with 1L-ALK-Inhibitor; 1L-ALKi (alectinib, ceritinib, crizotinib) 2L-ALKi second line ALK-inhibitor (change to different ALK-inhibitor than 1L). **(B)** Proposed algorithm for the management of patients with ALK-positive NSCLC and brain metastases under treatment with crizotinib. Postpone WBRT as long as possible.If BM not amenable for SRS switch to 2nd generation ALKi or if sparing WBI (2×20 Gy and/or hippocampal sparing) and TBP if later in the course of the disease. BM brain metastases; SRS stereotactic radiotherapy; WBRT whole brain radiotherapy; TBP treatment beyond progression with crizotinib; 2nd ALKi second generation ALK-inhibitor.

Figure 1 shows a proposed algorithm for the management of patients with brain metastases after first-line ALK-inhibitor therapy. Patients with an isolated progression of brain metastases should receive stereotactic radiotherapy. Treatment with TKI should be either continued (treatment beyond progression) - a treatment approach supported by Ou et al. [28] or switched to another ALK-inhibitor (based on detection of resistance mechanism like second site mutation or the development of other oncogenic drivers [93]). If patients with isolated brain metastasis cannot undergo stereotactic radiotherapy, further treatment could be either local therapy or switching to an alternative systemic therapy. The latter has become a valid option over WBRT due to the availability of the second generation ALK-inhibitors ceritinib, alectinib (and brigatinib, as soon as approved or within compassionate use programs). This must be accompanied by close imaging and clinical monitoring. While this approach can clearly be recommended in case of asymptomatic metastases, it may also be an option for some patients with symptomatic metastases. In addition, the time of progression may inform the decision for systemic or local therapy of CNS metastases. Progression of brain metastases early in the course of the disease would rather be an argument for switching to another ALK-inhibitor, since the potential neurocognitive side effects of radiotherapy might have a long-term impact on the patient's quality of life. Switching to a second ALK-inhibitor therapy should also be recommended in case of CNS metastases not amenable to stereotactic radiotherapy and coexistence of extracerebral metastases. It needs to be pointed out that these recommendations can support clinical decision-making in this particularly challenging situation, keeping in mind that they are not based on the data from prospective studies.
